# A Survey of 42 Semi-Volatile Organic Contaminants in Groundwater along the Grand Canal from Hangzhou to Beijing, East China

**DOI:** 10.3390/ijerph121215043

**Published:** 2015-12-18

**Authors:** Xiaojie Li, Zhu Rao, Zhipeng Yang, Xiaochen Guo, Yi Huang, Jing Zhang, Feng Guo, Chen Liu

**Affiliations:** National Research Center for Geoanalysis, Chinese Academy of Geological Sciences, Beijing 100037, China; lixiaojie99@126.com (X.L.); Pengpeng111x@163.com (Z.Y.); guoxc2012@163.com (X.G.); huangyi@126.com (Y.H.); jingzhang2015@163.com (J.Z.); binglingbeier@126.com (F.G.); liuchen0827@sohu.com (C.L.)

**Keywords:** trace organic contaminants, groundwater, East China, environmental risk assessment

## Abstract

The status of organic pollution in groundwater in eastern China along the Grand Canal from Hangzhou to Beijing was evaluated. Forty-two semi-volatile organic contaminants were analyzed, including 16 polycyclic aromatic hydrocarbons (PAHs), seven polychlorinated biphenyls (PCBs), 12 organochlorine pesticides (OCPs) and seven organophosphorus pesticides (OPPs). Among the detected contaminants, PAHs were the most widespread compounds. One PCB and six OCPs were detected in the groundwater samples, but none of the target OPPs was detected. The total concentration of the 16 PAHs ranged from 0.21 to 1006 ng/L, among which phenanthrene (271 ng/L) and fluoranthene (233 ng/L) were present at very high concentrations and naphthalene (32 positive detections in 50 samples) and fluorene (28 detections in 50 samples) were the most frequently detected. Benzo[a]pyrene equivalents indicated a high environmental risk related to PAHs in a few groundwater samples. To identify the possible sources of PAHs, three concentration ratios, low molecular weight PAHs/high molecular weight PAHs, anthracene/(anthracene + phenanthrene) and fluoranthene/(fluoranthene + pyrene), were determined, that indicated that the PAHs mainly originated from mixed sources: pyrolytic and petrogenic sources with different ratios at different sites.

## 1. Introduction

Groundwater, used mostly for drinking, agriculture and industry, plays a vital role in human survival, economic development and social harmony in China [1]. In general, groundwater is less contaminated by physical, chemical and biological pollution than surface waters due to its deeply buried environment [2]. However, due to rapid urbanization and frequent discharges of untreated wastewater to surface waters, freshwater is widely contaminated by man-made chemicals, which may result in groundwater pollution. Groundwater may suffer pollution from many sources. For example, groundwater in urban areas is likely to be impacted by pollutants from sewage, industrial activities, and diffuse leakages from reticulated sewerage and septic systems [3], while the main contributors to groundwater pollution in rural areas are probably fertilizers, agrochemicals, veterinary medicines related to agriculture and animal wastes [4]. Currently, increasing numbers of studies are detecting a large range of semi-volatile organic contaminants in groundwater [5,6,7].

East China has a long history of high population density and developed industry, and groundwater pollution is worsening due to the rapid development of society and the economy. In most rural areas, untreated groundwater is used directly for drinking water, agricultural irrigation and domestic water. Therefore, monitoring the quality of groundwater is essential. This study focuses on the status of trace organic contaminants in groundwater in the countryside of East China.

Polycyclic aromatic hydrocarbons (PAHs), polychlorinated biphenyls (PCBs), organochlorine pesticides (OCPs) and organophosphorus pesticides (OPPs) are the main organic contaminants in the environment, and are of significant concern due to their ubiquity and potential toxicity, persistence, and bioaccumulation [8]. PAHs, primarily formed during fossil fuel and biomass combustion and released in crude oil, petroleum and coal tar products, are one of the most widespread persistent organic pollutants in the environment [9]. Because of their carcinogenic and mutagenic properties, the U.S. Environmental Protection Agency has included 16 PAHs in their priority control list of persistent organic pollutants [10,11]. PCBs are synthetic chemicals that have been produced on an industrial scale since 1929 [12]. They are present in the environment mostly as mixtures that include various amounts of PCB congeners [13]. PCBs can resist physical, chemical, and biological degradation and have been detected in all the environments on Earth. The Stockholm Convention has listed PCBs as one of 12 persistent organic pollutants (POPs) [14].

Pesticides are widely used in agriculture in China. Hundreds of pesticides, including organochlorine, organophosphorus and carbamate insecticides, synthetic herbicides and fungicides are commonly used [15]. OCPs are man-made insecticides, fungicides, and anti-microbial chemicals. Nine of the twelve most hazardous POPs targeted by the Stockholm Convention in 2001 are OCPs. Organochlorine insecticides are potentially toxic, highly persistent, and resistant to biodegradation and it readily accumulates in human body tissues, causing a variety of health hazards. OCPs were widely used in China from 1950 to 1983 because of their low cost, high efficiency and broad-spectrum properties [16]. After use of OCPs was banned, OPPs became the most widely used pesticides because they exhibit moderate environmental persistence despite their high toxicity [17]. The research objectives of this study were: (1) to investigate the concentrations and distribution of 42 semi-volatile organic contaminants in groundwater near polluted area; (2) to assess the environmental risks of PAHs in groundwater and (3) to identify the potential sources of the main contaminants.

## 2. Materials and Methods

### 2.1. Study Area and Sampling

**Figure 1:**
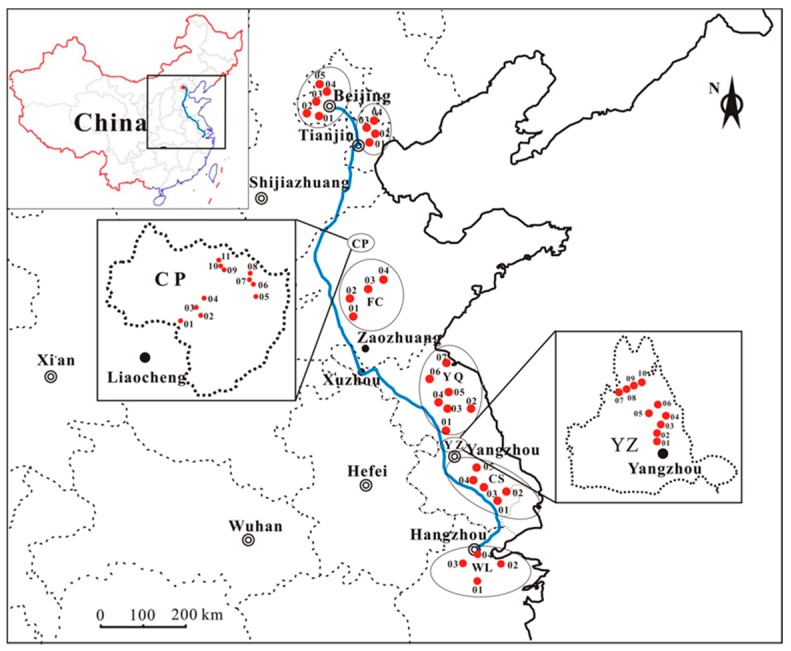
Sampling sites along the Grand Canal from Hangzhou to Beijing, East China.

The research area is located in Eastern China, where the economy is highly developed. Eight sites along the Grand Canal from Hangzhou to Beijing were selected as the sampling areas (Figure 1): Wuli (WL), Changshu (CS), Yangzhou (YZ), Yangqiao (YQ), Feicheng (FC), Chiping (CP), Beichen (BC) and Tongzhou (TZ). Wuli belongs to Zhejiang Province; Changshu, Yangzhou, and Yangqiao are located in Jiangsu Province; Feicheng and Chiping belong to Shandong Province; Beichen and Tongzhou are located in Tianjin and Beijing, respectively.

During June and July of 2014, fifty groundwater wells were investigated for organic contaminant analysis. The groundwater samples were stored in new amber glass bottles. Following collection, samples were immediately stored in a box containing ice and transported to the laboratory as soon as possible. The physical characteristics (Table 1) of the groundwater samples, including the number (No), site, sampling time, well depth, sampling depth, utilization, temperature (T), pH and redox potential (Eh), were measured *in situ*. All samples are arranged from south to north by latitude, and the well depth gradually increases from south to north. 

**Table 1:** Physicochemical properties of the groundwater samples.

No.	Sample Sites	Provinces	Sampling Time	Well Depth (m)	Sampling Depth (m)	Utilization	Temperature (°C)	pH	Eh (mV)
1	WL-01	Zhejiang	23 June 2014	5	2	Domestic water	--	--	--
2	WL-02	Zhejiang	23 June 2014	6	3	Agricultural	21.5	7.18	--
3	WL-03	Zhejiang	23 June 2014	4	1.5	Agricultural	22.8	7.11	--
4	WL-04	Zhejiang	23 June 2014	10	5	Agricultural	20.0	7.37	--
5	CS-01	Jiangsu	24 June 2014	5	2	Domestic water	19.9	7.33	--
6	CS-02	Jiangsu	24 June 2014	4.5	2	Domestic water	19.0	6.64	
7	CS-03	Jiangsu	24 June 2014	5	2	Domestic water	19.3	7.21	−7.0
8	CS-04	Jiangsu	24 June 2014	3	2	Domestic water	20.8	6.88	+8.8
9	CS-05	Jiangsu	24 June 2014	4	2.5	Domestic water	20.3	6.7	+20.7
10	YZ-01	Jiangsu	26 June 2014	23	--	Domestic water	18.8	6.7	18.9
11	YZ-02	Jiangsu	26 June 2014	4	1	Domestic water	23.0	7.23	−5.1
12	YZ-03	Jiangsu	26 June 2014	5	1.5	Domestic water	20.6	6.77	+22.9
13	YZ-04	Jiangsu	26 June 2014	4	1	Domestic water	21.6	7.34	−16.5
14	YZ-05	Jiangsu	26 June 2014	4.5	1	Domestic water	19.2	6.92	+12.0
15	YZ-06	Jiangsu	26 June 2014	6	2	Domestic water	20.7	6.92	+5.0
16	YZ-07	Jiangsu	26 June 2014	--	--	--	18.0	6.7	--
17	YZ-08	Jiangsu	26 June 2014			Domestic water	18.4	6.75	17.3
18	YZ-09	Jiangsu	26 June 2014	5	2	Domestic water	20.7	7.26	−20.4
19	YZ-10	Jiangsu	26 June 2014	5	1	Domestic water	21.2	7.41	−25.5
20	YQ-01	Jiangsu	28 June 2014		2.5	Domestic water	23.7	6.89	+4.3
21	YQ-02	Jiangsu	28 June 2014	26	--	Domestic water	18	6.74	+14.6
22	YQ-03	Jiangsu	28 June 2014	--	--	Domestic water	18.5	6.08	+47.1
23	YQ-04	Jiangsu	28 June 2014	--	--	--	--	--	--
24	YQ-05	Jiangsu	28 June 2014	--	4	Domestic water	17.8	6.24	+49.6
25	YQ-06	Jiangsu	28 June 2014	30	--	Domestic water	16.2	6.56	+56.5
26	YQ-07	Jiangsu	28 June 2014	29	--	Domestic water	18.0	7.17	−30.8
27	FC-01	Shandong	1 July 2014	--	--	--	--	--	--
28	FC-02	Shandong	1 July 2014	28	--	Domestic water	16.9	6.67	+19.5
29	FC-03	Shandong	1 July 2014	23	--	Domestic water	17.5	6.93	+1.0
30	FC-04	Shandong	1 July 2014	12	--	Domestic water	15.7	7.05	−28.0
31	CP-01	Shandong	3 July 2014	30	--	Domestic water	18.6	6.67	+21.5
32	CP-02	Shandong	3 July 2014	30	--	Domestic water	17.6	6.56	+27.0
33	CP-03	Shandong	3 July 2014	30	--	Domestic water	17.7	6.99	+14.6
34	CP-04	Shandong	3 July 2014	26	--	Domestic water	20.5	6.34	+39.1
35	CP-05	Shandong	3 July 2014	30	--	Domestic water	17.7	6.43	+34.5
36	CP-06	Shandong	3 July 2014	--	--	Domestic water	16.1	6.28	+42.5
37	CP-07	Shandong	3 July 2014	20	--	Domestic water	18.4	6.23	+33.2
38	CP-08	Shandong	3 July 2014	25	--	--	16.1	6.45	+33.4
39	CP-09	Shandong	3 July 2014	--	--	--	19.0	6.48	+31.0
40	CP-10	Shandong	3 July 2014	--	--	--	16.5	6.62	+21.9
41	CP-11	Shandong	3 July 2014	--	--	--	16.5	6.65	+21.4
42	BC-01	Tianjin	5 July 2014	100	--	Urban water	24.7	7.79	−46.8
43	BC-02	Tianjin	5 July 2014	100	--	Urban water	21.1	7.38	--
44	BC-03	Tianjin	5 July 2014	100	--	Urban water	23.4	7.2	−7.8
45	BC-04	Tianjin	5 July 2014	100	--	Urban water	24.2	7.66	−38.7
46	TZ-01	Beijing	6 July 2014	--	--	Agricultural	--	--	--
47	TZ-02	Beijing	6 July 2014	--	--	Agricultural	--	--	--
48	TZ-03	Beijing	6 July 2014	--	--	Agricultural	--	--	--
49	TZ-04	Beijing	6 July 2014	--	--	Domestic water	--	--	--
50	TZ-05	Beijing	6 July 2014	--	--	Domestic water	--	--	--

Note: “--”stands for not determined.

According to the Chinese Standards for Drinking Water Quality, the pH of drinking water should be between 6.5 and 8.5. Most of the samples complied with this standard except YQ-03 (6.08), YQ-05 (6.24), CP-04 (6.34), CP-05 (6.43), CP-06 (6.28), CP-07 (6.23), CP-08 (6.45) and CP-09 (6.48). The pH at these sites was lower than the standard, but the deviations were relatively small.

### 2.2. Chemicals

A mixture of sixteen U.S.EPA priority PAHs, acenaphthene (ANA), acenaphthylene (ANY), anthracene (ANT), benz[a]anthracene (BaA), benzo[a]pyrene (BaP), benzo[b]fluoranthene (BbF), benzo[g,h,i]perylene (BPE), benzo[k]fluoranthene (BKF), chrysene (CHR), dibenz[a,h]anthracene (DBA), fluoranthene (FLT), fluorene (FLU), indeno[1,2,3-cd]pyrene (IPY), naphthalene (NAP), phenanthrene (PHE) and pyrene (PYR) were purchased from AccuStandard ( New Haven, CT, USA) and a mixture of eight organochlorine pesticides: 1-chloro-2-[2,2,2-trichloro-1-(4-chlorphenyl)ethyl]benzene (o,p′-DDT), 1,1-dichloro-2,2-bis(*p*-chlorophenyl)ethane (p,p′-DDD), 1,1,1-trichloro-2,2-bis(4-chlorophenyl)ethane (p,p′-DDT), 2,2-dichloro-1,1-bis(4-chlorophenyl)ethane (p,p′-DDE), α-hexachlorocyclohexane (α-BHC), β-hexachlorocyclohexane (β-BHC), δ-hexachloro-cyclohexane (δ-BHC) and γ-hexachlorocyclohexane (γ-BHC), were purchased from o2si (Charleston, SC, USA). A mixture of seven PCBs: 2,4,4′-trichlorobiphenyl (PCB28), 2,2′,5,5′-tetrachloro-biphenyl (PCB52), 2,2′,4,5,5′-pentachlorobiphenyl (PCB101), 2,3′,4,4′,5-pentachlorobiphenyl (PCB118), 2,2′,3,4,4′,5′-hexachlorobiphenyl (PCB138), 2,2′,4,4′,5,5′-hexachlorobiphenyl (PCB153), 2,2′,3,4,4′,5,5′-heptachlorobiphenyl (PCB180), and eleven single standards of α-chlordane, γ-chlordane, bromophos, diazinon, fenitrothion, hexachlorobenzene, heptachlor, iprobenfos, isocarbophos, parathion-methyl and phenthoate were purchased from Ehrenstorfer GmbH (Augsburg, Germany). Two surrogate standards, 2,4,5,6-tetrachloro-*m*-xylene and dibutyl chlorendate and four internal standards, acenaphthene-d_10_, chrysene-d_12_, perylene-d_12_ and phenanthrene-d_10_, were also obtained from Ehrenstorfer GmbH.

### 2.3. Sample Pretreatment and Instrumental Analysis 

The details of the sample pretreatment and instrumental analysis have been described in a previous publication [18]. Briefly, the groundwater samples were extracted using liquid-liquid extraction. First, 1 L of groundwater was added into a separatory funnel along with 30.0 g of sodium chloride. Then, the water samples were successively extracted three times with 50, 25 and 25 mL of extraction solvent (a 4:1 v:v mixture of *n*-hexane and ethyl acetate); each extraction was shaken for 10 min. Finally, the organic phases were concentrated to 1 mL. The target compounds were then analyzed on a Thermo Scientific GCMS-Trace GC Ultra instrument (Waltham, MA, USA) equipped with an electron impact (EI) ionization source in selected ion monitoring (SIM) mode. A DB-5MS fused silica capillary column (30 m length × 0.25 mm diameter × 0.25 μm film thickness) was used for the separation of the target compounds. Samples (1 μL) were injected in splitless mode. The carrier gas was helium at a flow rate of 1.2 mL/min. The injector and mass transfer temperatures were maintained at 265 °C and 290 °C, respectively. The temperature program was as follows: 60 °C (held for 1 min), ramped to 160 °C at a rate of 18 °C/min and then ramped to 320 °C at 8 °C/min (held for 10 min). 

## 3. Results and Discussion

### 3.1. Status and Occurrence of 42 Semi-Volatile Organic Contaminants

The concentrations of 42 semi-volatile organic contaminants in groundwater samples from East China were in the range of 0.17–271 ng/L. Overall, the highest concentrations detected were of phenanthrene (271 ng/L), fluoranthene (233 ng/L), naphthalene (188 ng/L), pyrene (131 ng/L), fluorene (63.1 ng/L) and anthracene (58.6 ng/L). Other than the concentration of naphthalene in Jiangsu Province, the rest top five compounds (in terms of concentration) were detected in Shandong Province, which perhaps indicates that pollution is more serious in Shandong and Jiangsu Province. In Zhejiang, Tianjin and Beijing, the maximum concentrations were much lower. Nineteen of the forty-two targets were not detected, including six polychlorinated biphenyls (PCBs), six organochlorine pesticides (OCPs) and seven organophosphorus pesticides (OPPs).

The detected number (DN) and minimum, mean and maximum concentrations at the Zhejiang, Jiangsu, Shandong, Tianjin and Beijing Provinces are shown in Table 2. In Zhejiang Province 13 of 42 compounds were detected, including 12 PAHs and one pesticide (hexachlorobenzene). Hexachlorobenzene had been detected in all of samples (*n* = 4), but at very low concentration. In Jiangsu Province 14 PAHs, one PCB and six pesticides were detected. The most frequently detected PAHs were naphthalene (17 positives in 22 samples), benzo[a] pyrene (12 detections in 22 samples) and fluoranthene (11 detections in 22 samples), and the most frequently detected pesticide was hexachlorobenzene (9 detections in 22 samples). In Shandong, Tianjin, and Beijing, only PAHs were detected. In Shandong Province fluorene (11 detections in 15 samples) and acenaphthene (9 detections in 15 samples) were the most frequently detected PAHs. Naphthalene (3 detections in 4 samples) and benzo[a] pyrene (3 detections in 4 samples) were the most frequently detected pollutants in Tianjin Province. In Beijing naphthalene, acenaphthylene, acenaphthene, phenanthrene, anthracene, fluoranthene, pyrene, and benzo[b]-fluoranthene (all with four positive detections in five samples) were the most frequently detected PAHs. Overall, the total 42 compounds had comparable detection frequencies at all of the sites, but only one PCB (PCB28) was detected in Jiangsu Province. Naphthalene (32 detections in 50 samples) and fluorene (28 in 50 samples) were the most frequently detected compounds in the all groundwater samples.

**Table 2:** Method detection limits (MDL), detection number (DN) and concentrations of detection.

CAS Number	Name	MDL ng/L	Total Sites (*n* = 50)	Zhejiang Province (*n* = 4)				Jiangsu Province (*n* = 22)				Shandong Province (*n* = 15)				Tianjin (*n* = 4)				Beijing (*n* = 5)			
			DN	DN	Min	Mean	Max	DN	Min	Mean	Max	DN	Min	Mean	Max	DN	Min	Mean	Max	DN	Min	Mean	Max
					ng/L				ng/L				ng/L				ng/L				ng/L		
***PAHs***																							
91-20-3	Naphthalene	0.23	32	3	50.9	70.0	89.7	17	7.04	69.0	188	5	15.1	56.2	103	3	36.7	62.8	95.6	4	3.95	21.9	44.7
208-96-8	Acenaphthylene	0.35	18	1	1.87	1.87	1.87	6	1.40	6.33	22.7	6	0.44	2.62	5.44	1	11.0	11.0	11.0	4	0.52	2.91	9.34
83-32-9	Acenaphthene	0.30	25	3	1.68	4.23	8.51	9	0.58	2.84	8.37	9	0.23	10.8	83.4	1	6.40	6.40	6.40	4	0.70	8.23	29.1
86-73-7	Fluorene	0.17	28	2	2.79	5.13	7.48	10	0.17	8.10	42.1	11	1.07	13.2	63.1	2	1.11	22.5	43.9	3	2.99	11.5	27.5
85-01-8	Phenanthrene	0.10	20	2	4.56	11.5	18.4	8	0.75	25.1	57.0	4	11.1	101	271	2	3.64	60.2	117	4	1.04	18.0	61.0
120-12-7	Anthracene	0.24	21	2	5.78	8.00	10.2	8	2.85	10.4	28.0	5	2.01	23.7	58.6	2	5.69	20.4	35.1	4	1.61	4.12	8.97
206-44-0	Fluoranthene	0.29	23	2	3.74	21.9	40.1	11	1.34	16.2	39.1	4	11.3	141	233	2	15.5	39.2	64.3	4	1.81	6.72	14.8
129-00-0	Pyrene	0.29	20	2	0.42	24.1	47.7	9	2.31	19.4	41.2	4	10.7	74.3	131	1	32.3	32.3	32.3	4	3.05	5.00	9.34
56-55-3	Benzo[a]anthracene	0.20	1	0	<0.20	<0.20	<0.20	0	<0.20	<0.20	<0.20	1	40.6	40.6	40.6	0	<0.20	<0.20	<0.20	0	<0.20	<0.20	<0.20
218-01-9	Chrysene	0.20	1	0	<0.20	<0.20	<0.20	0	<0.20	<0.20	<0.20	1	45.3	45.3	45.3	0	<0.20	<0.20	<0.20	0	<0.20	<0.20	<0.20
205-99-2	Benzo[b]fluoranthene	0.17	13	1	38.3	38.3	38.3	5	0.38	2.07	3.66	3	1.34	24.7	42.3	0	<0.17	<0.17	<0.17	4	0.58	1.10	2.17
207-08-9	Benzo[k]fluoranthene	0.20	11	0	<0.20	<0.20	<0.20	8	0.21	1.27	4.26	3	0.77	9.39	15.5	0	<0.20	<0.20	<0.20	0	<0.20	<0.20	<0.20
50-32-8	Benzo[a]pyrene	0.29	20	0	<0.29	<0.29	<0.29	12	0.48	1.65	4.02	3	1.63	18.46	28.85	3	0.35	0.75	1.54	2	0.95	1.05	1.15
193-39-5	Indeno[1,2,3-cd]pyrene	0.20	12	2	0.52	3.55	6.59	8	0.24	1.15	4.12	2	11.0	16.8	22.6	0	<0.20	<0.20	<0.20	0	<0.20	<0.20	<0.20
53-70-3	Dibenzo[a,h]anthracene	0.74	8	1	2.04	2.04	2.04	5	0.43	2.58	4.69	2	3.69	3.98	4.26	0	<0.74	<0.74	<0.74	0	<0.74	<0.74	<0.74
191-24-2	Benzo[g,h,i]perylene	0.23	15	2	4.64	6.33	8.02	10	0.27	1.10	3.79	3	0.34	11.39	21.71	0	<0.23	<0.23	<0.23	0	<0.23	<0.23	<0.23
***PCBs***																							
7012-37-5	PCB 28	0.23	3	0	<0.23	<0.23	<0.23	3	0.35	1.51	2.44	0	<0.23	<0.23	<0.23	0	<0.23	<0.23	<0.23	0	<0.23	<0.23	<0.23
35693-99-3	PCB 52	0.40	0	0	<0.40	<0.40	<0.40	0	<0.40	<0.40	<0.40	0	<0.40	<0.40	<0.40	0	<0.40	<0.40	<0.40	0	<0.40	<0.40	<0.40
37680-73-2	PCB 101	0.50	0	0	<0.50	<0.50	<0.50	0	<0.50	<0.50	<0.50	0	<0.50	<0.50	<0.50	0	<0.50	<0.50	<0.50	0	<0.50	<0.50	<0.50
31508-00-6	PCB 118	0.17	0	0	<0.17	<0.17	<0.17	0	<0.17	<0.17	<0.17	0	<0.17	<0.17	<0.17	0	<0.17	<0.17	<0.17	0	<0.17	<0.17	<0.17
35065-28-2	PCB 138	0.41	0	0	<0.41	<0.41	<0.41	0	<0.41	<0.41	<0.41	0	<0.41	<0.41	<0.41	0	<0.41	<0.41	<0.41	0	<0.41	<0.41	<0.41
35065-27-1	PCB 153	0.45	0	0	<0.45	<0.45	<0.45	0	<0.45	<0.45	<0.45	0	<0.45	<0.45	<0.45	0	<0.45	<0.45	<0.45	0	<0.45	<0.45	<0.45
35065-29-3	PCB 180	0.50	0	0	<0.50	<0.50	<0.50	0	<0.50	<0.50	<0.50	0	<0.50	<0.50	<0.50	0	<0.50	<0.50	<0.50	0	<0.50	<0.50	<0.50
***Pesticides ***																							
118-74-1	Hexachlorobenzene	0.10	13	4	0.23	0.72	1.54	9	0.1	5.84	18.3	0	<0.10	<0.10	<0.10	0	<0.10	<0.10	<0.10	0	<0.10	<0.10	<0.10
319-84-6	α-BHC	0.47	0	0	<0.47	<0.47	<0.47	0	<0.47	<0.47	<0.47	0	<0.47	<0.47	<0.47	0	<0.47	<0.47	<0.47	0	<0.47	<0.47	<0.47
319-85-7	β-BHC	0.93	0	0	<0.93	<0.93	<0.93	0	<0.93	<0.93	<0.93	0	<0.93	<0.93	<0.93	0	<0.93	<0.93	<0.93	0	<0.93	<0.93	<0.93
319-86-8	γ-BHC	0.72	0	0	<0.72	<0.72	<0.72	0	<0.72	<0.72	<0.72	0	<0.72	<0.72	<0.72	0	<0.72	<0.72	<0.72	0	<0.72	<0.72	<0.72
58-89-9	δ-BHC	0.91	0	0	<0.91	<0.91	<0.91	0	<0.91	<0.91	<0.91	0	<0.91	<0.91	<0.91	0	<0.91	<0.91	<0.91	0	<0.91	<0.91	<0.91
72-55-9	p,p´-DDE	0.30	7	0	<0.30	<0.30	<0.30	7	0.34	3.21	11.0	0	<0.30	<0.30	<0.30	0	<0.30	<0.30	<0.30	0	<0.30	<0.30	<0.30
72-54-8	p,p´-DDD	0.34	0	0	<0.34	<0.34	<0.34	0	<0.34	<0.34	<0.34	0	<0.34	<0.34	<0.34	0	<0.34	<0.34	<0.34	0	<0.34	<0.34	<0.34
789-02-6	o,p´-DDT	0.44	1	0	<0.44	<0.44	<0.44	1	3.38	3.38	3.38	0	<0.44	<0.44	<0.44	0	<0.44	<0.44	<0.44	0	<0.44	<0.44	<0.44
50-29-3	p,p´-DDT	0.31	1	0	<0.31	<0.31	<0.31	1	2.76	2.76	2.76	0	<0.31	<0.31	<0.31	0	<0.31	<0.31	<0.31	0	<0.31	<0.31	<0.31
5103-71-9	α-Chlordane	0.50	2	0	<0.50	<0.50	<0.50	2	0.73	1.08	1.43	0	<0.50	<0.50	<0.50	0	<0.50	<0.50	<0.50	0	<0.50	<0.50	<0.50
5103-74-2	γ-Chlordane	0.46	2	0	<0.46	<0.46	<0.46	2	0.48	0.69	0.90	0	<0.46	<0.46	<0.46	0	<0.46	<0.46	<0.46	0	<0.46	<0.46	<0.46
76-44-8	Heptachlor	0.63	0	0	<0.63	<0.63	<0.63	0	<0.63	<0.63	<0.63	0	<0.63	<0.63	<0.63	0	<0.63	<0.63	<0.63	0	<0.63	<0.63	<0.63
298-00-0	Parathion-methyl	2.67	0	0	<2.67	<2.67	<2.67	0	<2.67	<2.67	<2.67	0	<2.67	<2.67	<2.67	0	<2.67	<2.67	<2.67	0	<2.67	<2.67	<2.67
122-14-5	Fenitrothion	3.08	0	0	<3.08	<3.08	<3.08	0	<3.08	<3.08	<3.08	0	<3.08	<3.08	<3.08	0	<3.08	<3.08	<3.08	0	<3.08	<3.08	<3.08
24353-61-5	Isocarbophos	0.74	0	0	<0.74	<0.74	<0.74	0	<0.74	<0.74	<0.74	0	<0.74	<0.74	<0.74	0	<0.74	<0.74	<0.74	0	<0.74	<0.74	<0.74
2104-96-3	Bromophos	1.54	0	0	<1.54	<1.54	<1.54	0	<1.54	<1.54	<1.54	0	<1.54	<1.54	<1.54	0	<1.54	<1.54	<1.54	0	<1.54	<1.54	<1.54
333-41-5	Diazinon	2.93	0	0	<2.93	<2.93	<2.93	0	<2.93	<2.93	<2.93	0	<2.93	<2.93	<2.93	0	<2.93	<2.93	<2.93	0	<2.93	<2.93	<2.93
26087-47-8	Iprobenfos	1.77	0	0	<1.77	<1.77	<1.77	0	<1.77	<1.77	<1.77	0	<1.77	<1.77	<1.77	0	<1.77	<1.77	<1.77	0	<1.77	<1.77	<1.77
2597-03-7	Phenthoate	1.49	0	0	<1.49	<1.49	<1.49	0	<1.49	<1.49	<1.49	0	<1.49	<1.49	<1.49	0	<1.49	<1.49	<1.49	0	<1.49	<1.49	<1.49

### 3.2. Environmental Risks and Sources of PAHs

**Figure 2:**
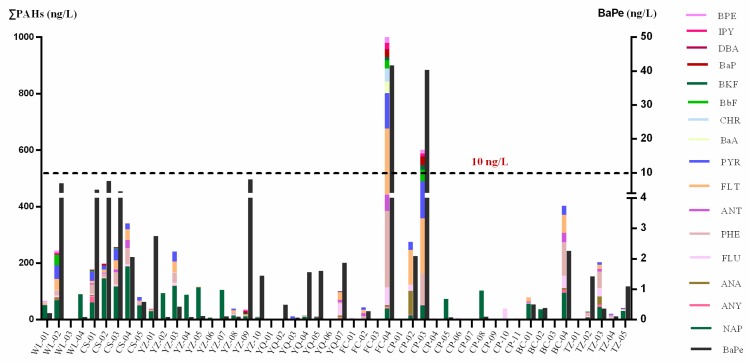
Concentrations and benzo[a]pyrene equivalent concentrations of PAHs in the groundwater samples.

PAHs were the main contaminants in the groundwater samples. Different from PCBs, OCPs and OPPs, almost all of the groundwater samples contained PAHs. Figure 2 shows the concentrations of different PAHs in all of the samples. The total concentrations of PAHs in groundwater ranged from 0.21 to 1006 ng/L. The highest concentration was detected in FC-04 (1006 ng/L) followed by CP-03 (606 ng/L), both of which were located in Shandong Province.

Because of the relatively large amount of toxicological data available on BaP, this compound was chosen as the primary representative of the PAH class [19]. In 1992, LaGoy and Nisbet presented a toxic equivalency factor (TEF) scheme for 17 PAHs. Along similar lines, the Office of Environmental Health Hazard Assessment (OEHHA) of the California EPA has developed a potency equivalency factor (PEF) procedure to assess the relative potencies of PAHs and PAH derivatives as a group for California’s Toxic Air Contaminant program [20,21]. This procedure can be used to assess the impact of carcinogenic PAHs in ambient air, in which several PAHs are usually present together. In this study, benzo[a]pyrene equivalent concentrations (BaPe) were also used to assess the environmental risks of PAHs in groundwater samples [22,23] and are obtained by multiplying the individual PAH concentration by its potency equivalency factor (PEF) via the following Equation (1):

(1) Bape=∑(CPAHi×PEFi)

where BaPe is the sum of benzo[a]pyrene equivalent concentrations of the 16 detected PAHs, CPAHi represents the concentrations of multi-component PAHs and PEFi represents the potency equivalency factors of the studied PAHs, which are provided in Table 3.

The BaPe values at all of the sites (Figure 2) ranged from 0.0003 ng/L to 41.7 ng/L, with a mean value of 3.04 ng/L. Among the 50 sites with detections, only FC-04 and CP-03 had BaPe values that were greater than the national standard of China (10 ng/L, GB 5749-2006). However, the BaPe values at these sites were higher than 40.0 ng/L, indicating environmental health risks at these locations.

**Table 3:** Potency equivalency factors (PEF) for individual PAHs relative to BaP used in this study.

Compounds	PEF_i_
Benzo[a]pyrene, dibenz[a, h]anthracene	1
Benzo[a]anthracene, benzo[b]fluoranthene, benzo[k]fluoranthene, indeno[1,2,3-c, d]pyrene	0.1
Anthracene, chrysene, benzo(g,h,i)perylene	0.01
Naphthalene, acenaphthylene, acenaphthene, fluorene, phenanthrene, fluoranthene, pyrene	0.001

**Figure 3:**
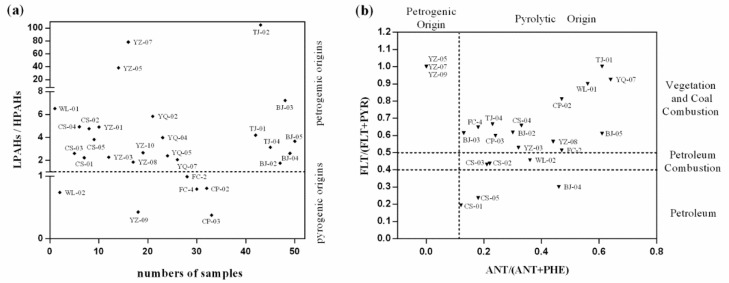
Sources identification: (**a**) concentration ratios of LPAHs/HPAHs; and (**b**) (ANT/(ANT + PHE) *vs.* FLT/(FLT + PYR).

Identification of the possible sources of PAHs is important to understanding the fate of PAHs in the environment. In general, PAHs mainly originate from pyrolytic sources (incomplete combustion of petroleum, vegetation and coal) and petrogenic sources (production, transport and use of petroleum and its refined products) [9]. Petrogenic sources are enriched by low molecular weight PAHs (LPAHs, 2–3 rings), whereas pyrolytic sources are dominated by high molecular weight PAHs (HPAHs, 4–6 rings). Thus, concentration ratios of ∑LPAHs/∑HPAHs can be used to determine the petrogenic and pyrolytic sources of PAHs in groundwater. According to previous studies, ∑LPAHs/∑HPAHs > 1 indicates petrogenic origins, and ∑LPAHs/∑HPAHs < 1 signals pyrogenic origins [24]. According to the ∑LPAHs/∑HPAHs ratios in Figure 3a, PAHs in WL-01 are possibly of petrogenic origin, but those in WL-02 possibly have a pyrogenic origin. 

In Jiangsu Province, almost all the sites may have petrogenic sources except for YZ-09, which is the same finding as in Tianjin and Beijing. Pyrogenic sources are likely in Shandong Province. In addition, the concentration ratios of some isomers have been used as indices of the origin of PAHs [11,25]. Doong and Lin used PHE/ANT, FLT/PYR and CHR/BaA to evaluate the possible sources of PAHs [25]. Similarly, Bing Wu used ANT/(ANT + PHE) and FLT/(FLT + PYR) as indices to interpret PAH components and deduce their possible sources [9]. PHE/ANT has same meaning as ANT/(ANT + PHE), and FLT/PYR has the same meaning as FLT/(FLT + PYR). In the present study, we selected ANT/(ANT + PHE) and FLT/(FLT + PYR) to evaluate the possible PAH sources. The scatterplot of FLT/(FLT + PYR) and ANT/(ANT + PHE) shows both of these ratios at all the sites (Figure 3b). The majority of the sites had FLT/(FLT + PYR) ratios higher than 0.4, except at CS-01, CS-05 and BJ-04, and most sites had ANT/(ANT + PHE) ratios higher than 0.1, except for YZ-05, YZ-07, and YZ-09, because phenanthrene and anthracene were not detected at these latter three sites. From the congener-pair ratios, the sources of PAHs in the groundwater of eastern China might be the combustion of vegetation, coal and petroleum. Our results are consistent with other literature on the sources of PAHs in China [9,24]. Overall, we can conclude from these different ratios that the PAHs in the groundwater of eastern China predominantly originate from composite sources.

### 3.3. PCBs and Pesticides in Groundwater Samples

#### 3.3.1. Polychlorinated Biphenyls (PCBs) 

**Figure 4:**
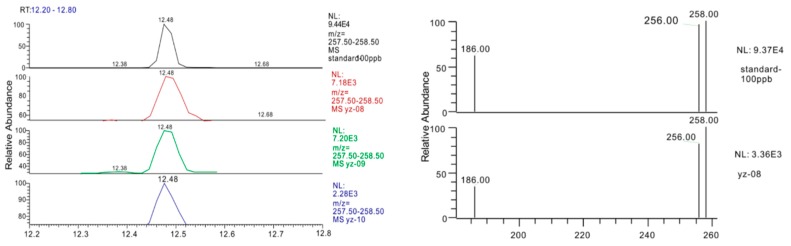
Comparison of chromatograms and mass spectra of PCB28 in a standard (100 ng/mL) and the samples.

Polychlorinated biphenyls (PCBs) are listed by the Stockholm Convention as widespread persistent organic pollutants with high bioaccumulation, toxicity, long term persistency, that can cause environmental damage and represent an important class of priority pollutants, although their uses have been banned in a lot of countries. In China, approximately 10,000 tons of PCBs were produced from 1965 to 1974 [26]. In Table 2, only one of the seven PCBs, PCB28, was detected at YZ-08 (1.73 ng/L), YZ-09 (2.44 ng/L) and YZ-10 (0.35 ng/L) of Jiangsu Province. Figure 4 shows the mass chromatogram of PCB28 (*m/z* 258) in the three positive samples and a standard (100 ng/mL) and compares the mass spectra between the standard and samples, demonstrating a high good alignment of retention time and fragment ions. PCB28 belong to the trichlorobiphenyl group which was mainly used for capacitor production [27]. The improper disassembly of polychlorinated biphenyl (PCB)-containing equipment such as capacitors and electrical transformers may lead to PCB pollution [27]. Considerable attention should be paid to the migration of low chlorinated PCBs in soil to groundwater.

#### 3.3.2. Pesticides

Despite the ban on OCPs in China in 1983, these compounds can still be detected in various environmental media. In China, there have been many studies of OCP residues in soils [28,29,30], sediments [31,32] and surface waters [33,34]. However, there is little information about OCPs in groundwater [23]. This study provides the OCP pollution status in groundwater of East China. Among the 12 target organochlorine pesticides, hexachlorobenzene, p,p′-DDE, p,p′-DDT, α-chlordane and γ-chlordane were detected in our groundwater samples. Technical DDT generally contains p,p′-DDT (75%), o,p′-DDT (15%) and p,p′-DDE (5%), and DDT can be degraded to DDE and DDD in aerobic and anaerobic environments, respectively [35]. In this study, DDT was only present at YZ-02 at 2.76 and 3.38 ng/L (p,p′-DDT and o,p′-DDT, respectively); in the same sample, the concentration of p,p′-DDE was 11.04 ng/L. p,p′-DDE also present in the predominant percentage in the other six samples with positive detections, demonstrating that DDT had degraded to DDE in these aerobic environments. Hexachlorobenzene was the most frequently detected OCP in this study, with concentrations ranging from 0.10 to 1.54 ng/L, and α- and γ-chlordane were only found in YZ-08 and YZ-09 at low concentrations. Organophosphorous pesticidesare known to degrade rapidly depending on their formulation, method of application, climate and the growing stage of the plant [36]. None of the detected seven OPPs was found in the total groundwater samples, this may be due to their property of moderate environmental persistence.

## 4. Conclusions

This work investigated the contamination status of 42 semi-volatile organic contaminants in groundwater in East China along the Grand Canal from Hangzhou to Beijing. PAHs, PCBs and OCPs detected in some of the groundwater samples, and PAHs were the most widely distributed organic pollutants. The PAHs detected in the groundwater of this study area primarily originated from a combination of pyrolytic and petrogenic sources. Ninety percent of the studied sites had lower benzo[a]pyrene equivalent concentrations than the accepted value (10 ng/L) recommended by China. However, specific sites such as FC-04 and CP-03, with BaPe values higher than 40.0 ng/L, should be given special attention. Despite the ban on OCPs in China in 1983, some OCPs were still detected in this study, but at low concentrations. Fortunately, only one of the seven target PCBs and none of the target OPPs were found in groundwater samples.
